# IBTK contributes to B-cell lymphomagenesis in *Eμ-myc* transgenic mice conferring resistance to apoptosis

**DOI:** 10.1038/s41419-019-1557-6

**Published:** 2019-04-11

**Authors:** Eleonora Vecchio, Gaetanina Golino, Antonio Pisano, Francesco Albano, Cristina Falcone, Simona Ceglia, Enrico Iaccino, Selena Mimmi, Giuseppe Fiume, Giorgio Giurato, Domenico Britti, Giuseppe Scala, Ileana Quinto

**Affiliations:** 1 Department of Experimental and Clinical Medicine, University Magna Græcia of Catanzaro, Catanzaro, 88100 Italy; 2 Laboratory of Molecular Medicine and Genomics, Department of Medicine, Surgery and Dentistry “Scuola Medica Salernitana”, University of Salerno, Baronissi, SA Italy; 3 Genomix4Life srl, Department of Medicine, Surgery and Dentistry “Scuola Medica Salernitana”, University of Salerno, Baronissi, SA Italy; 4 Department of Health Science, University Magna Græcia of Catanzaro, Catanzaro, 88100 Italy; 5 Interdepartmental Services Centre of Veterinary for Human and Animal Health, University “Magna Græcia” of Catanzaro, Catanzaro, 88100 Italy

## Abstract

Increasing evidence supports the involvement of IBTK in cell survival and tumor growth. Previously, we have shown that *IBTK* RNA interference affects the wide genome expression and RNA splicing in cell-type specific manner. Further, the expression of *IBTK* gene progressively increases from indolent to aggressive stage of chronic lymphocytic leukemia and decreases in disease remission after therapy. However, the role of IBTK in tumorigenesis has not been elucidated. Here, we report that loss of the murine *Ibtk* gene raises survival and delays tumor onset in *Eμ-myc* transgenic mice, a preclinical model of Myc-driven lymphoma. In particular, we found that the number of pre-cancerous B cells of bone marrow and spleen is reduced in *Ibtk*^*−/−*^*Eμ-myc* mice owing to impaired viability and increased apoptosis, as measured by Annexin V binding, Caspase 3/7 cleavage assays and cell cycle profile analysis. Instead, the proliferation rate of pre-cancerous B cells is unaffected by the loss of *Ibtk*. We observed a direct correlation between *Ibtk* and *myc* expression and demonstrated a Myc-dependent regulation of *Ibtk* expression in murine B cells, human hematopoietic and nonhematopoietic cell lines by analysis of ChIP-seq data. By tet-repressible Myc system, we confirmed a Myc-dependent expression of IBTK in human B cells. Further, we showed that *Ibtk* loss affected the main apoptotic pathways dependent on Myc overexpression in pre-cancerous *Eμ-myc* mice, in particular, MCL-1 and p53. Of note, we found that loss of *IBTK* impaired cell cycle and increased apoptosis also in a human epithelial cell line, HeLa cells, in Myc-independent manner. Taken together, these results suggest that *Ibtk* sustains the oncogenic activity of Myc by inhibiting apoptosis of murine pre-cancerous B cells, as a cell-specific mechanism. Our findings could be relevant for the development of *IBTK* inhibitors sensitizing tumor cells to apoptosis.

## Introduction

The human *IBTK* gene maps on the 6q14.1 genetic locus, a hotspot of chromosomal aberrations in lymphoproliferative disorders. IBtkα is the most abundant protein isoform, sharing a high homology with the murine Ibtk protein^1^. It has been functionally characterized as substrate receptor of Cullin 3 Ubiquitin ligase complex (CRL3^IBTK^) promoting the ubiquitination coupled to proteasomal degradation of Pdcd4, a translational inhibitor^2,3^. Silencing of *IBTK* by RNA interference in HeLa and K562 cells modified the wide genome expression and RNA splicing^4^. Altogether, these findings indicate that *IBTK* has pleiotropic effects, being involved in protein turnover and RNA metabolism.

Preliminary evidence supports the involvement of *IBTK* in cell survival upon cellular stress. Indeed, *IBTK* RNA interference promotes the apoptosis of murine embryonic fibroblasts treated with thapsigargin or tunicamycin, two inducers of endoplasmic reticulum stress^5^. Further, increased production of IBtkα occurs in human bronchial epithelial cells exposed to the industrial pollutant titanium dioxide, as part of stress cellular response^6^. Additional findings suggest the involvement of *IBTK* in tumorigenesis. *IBTK* RNA interference causes loss of viability of K-Ras-mutant colorectal cancer cells^7^. A different methylation pattern of the *IBTK* gene is reported in poor-prognostic Immunoglobulin Heavy Variable Chain (IGHV)-unmutated Chronic Lymphocytic Leukemia (U-CLL) compared with favorable prognostic IGHV-mutated CLL (M-CLL)^8^, suggesting that the altered *IBTK* expression could be associated with tumor progression and aggressiveness. Recently, we have shown a strict correlation between the up-regulation of *IBTKα* expression and CLL progression, conferring resistance to apoptosis in tumor B-cell lines^9^.

Consistently with these observations, *IBTK* could be required for B-cell lymphomagenesis. To address this question, we analyzed the impact of *IBTK* loss in the *Eμ-myc* transgenic mouse, a preclinical model of human Myc-driven lymphoma^10^.

c-Myc is a member of the basic helix-loop-helix–leucine zipper Myc transcription factors and regulates the expression of several genes involved in cell proliferation, differentiation, metabolism, cell growth and apoptosis^11,12^. The expression of c-Myc is tightly regulated at transcriptional, post-transcriptional and post-translational level^13–16^ and its deregulation occurs in several kinds of tumors^17^. Noteworthy, c-Myc is frequently overexpressed in hematological malignancies due to gene amplification or translocation^18,19^. The *Eμ-myc* transgenic mouse bears the *c-myc*/immunoglobulin gene translocation leading to overexpression of *c-myc* gene in B-cell lineage with development of aggressive pre-B and/or B-cell lymphomas with a median age of death at about 100 days^10,20,21^. Myc-driven lymphomas develop from B220^low^ pre-B and immature B-cell pools, and *Ig* gene rearrangement analyses indicate that most are monoclonal^10^.

In this study, we show that loss of the *Ibtk* gene in *Eμ-myc* transgenic mice delays the onset of B lymphoma and improves animal survival as consequence of increased apoptosis of pre-cancerous B cells. Our findings support the first evidence on pro-survival action of *IBTK* in Myc-driven B cells, providing the rationale for the development of novel therapeutic approaches of B lymphoma.

## Materials and methods

### Mice

Knockout of the murine *Ibtk* gene was obtained by using the XF224 embryonic stem (ES) cell line, which carries the gene trap vector pGT2Lxf from BayGenomics (http://www.genetrap.org/), randomly inserted within introns; pGT2Lxf contains a splice-acceptor sequence upstream of *βgeo* gene reporter, a fusion between *β−galactosidase* and *neomycin phosphotransferase II*^22^. The XF224 ES clone carries the *Ibtk* gene disrupted by insertional mutagenesis of pGT2Lxf within the intron 22. Knockout of *Ibtk* was determined by 5′ rapid amplification of cDNA ends followed by automated DNA sequencing (sequence information at http://www.informatics.jax.org/allele/MGI:4129389). For generating *Ibtk*^*−/−*^ mice, the XF224 ES clone was microinjected into C57BL/6 J blastocysts; the resulting male chimeras were mated with female C57BL/6 J mice and backcrossed for 8 generations. Heterozygous *Ibtk*^+/−^ offspring was inter-crossed to produce homozygous *Ibtk*^−/−^ mice.

*Eμ-myc* transgenic mice (TgN(IghMyc)22Bri/J) were obtained from The Jackson Laboratory (Bar Harbor, Maine; USA). Both *Eμ-myc* transgenic mice and *Ibtk*^*−/−*^ mice were congenic with C57BL/6 J mice. *Eμ-myc* transgenic mice were crossed with *Ibtk*^*−/−*^ mice to generate *Ibtk*^*+/−*^ *Eμ-myc* mice. The F1 offspring was crossed with *Ibtk*^*−/−*^ or *Ibtk*^*+/−*^ mice to generate *Ibtk*^*+/+*^ *Eμ-myc* and *Ibtk*^*−/−*^*Eμ-myc* littermates.

The *Eμ-myc* transgene was detected by genomic PCR amplification of 600-bp product as described^23^. Genotyping for *Ibtk* and *βgeo* genes was performed using the primers 5′-GATGTAAAGCCGTGGGAGAA-3′ and 5′-ATGTGGAGAGGAGGCAGAGA-3′ (792 bp product), and 5′-GATGTAAAGCCGTGGGAGAA-3′ and 5′-CACTCCAACCTCCGCAAACTC-3′ (550 bp product), respectively. Mice were daily monitored for signs of morbidity and tumor development. For pre-cancerous analysis, 4–6 weeks old mice with no infiltration of peripheral lymph nodes were used.

The experimental protocols have been approved by the Bioethical Committee of the University Magna Graecia of Catanzaro; the animal experiments were carried out in accordance with the protocol n.794/2016-PR approved by the Italian Ministry of Health.

### Peripheral blood cell counts

Blood was collected from the retro-orbital sinus of mice, as described^24^. Peripheral blood cells measured using ADVIA 2120 Hematological analyzer (Siemens Healthcare, Erlangen, Germany).

### Cell culture, reagents, plasmids, lentiviruses, and siRNA

Cells were cultured at 37°C in a humidified atmosphere containing 5% CO_2_. B cells, P493-6, HeLa cell lines were grown in completed Iscove's Modified Dulbecco's Medium, Roswell Park Memorial Institute (RPMI) 1640 and Dulbecco's Modified Eagle Medium (DMEM) medium (Thermo Fisher Scientific, Waltham, MA, USA) supplemented with 10% fetal bovine serum (FBS), 2 mm l-glutamine, 1 mm Na-pyruvate, and 50 mm 2-mercaptoethanol and 100U/ml penicillin, 100 μg/ml streptomycin; all reagents from Thermo Fisher Scientific. Separation of B cells was performed by depletion of non-B cells using magnetic-activated cell sorting (MACS) B-cell isolation kit or by CD19 MicroBeads and MS columns (Miltenyi Biotech, Bergisch Gladbach, Germany) according to the manufacturer’s protocols. Control flow cytometry from MACS separated cells revealed 95% purity.

For P493-6 cells, to turn-off MYC expression, cells were grown in the presence of 0.1 μg/ml of tetracycline (Sigma) for 72 h. P493-6 cells were treated with tetracycline for 72 h and then washed with RPMI1640 for three times to remove tetracycline, then the cells were maintained in RPMI1640 with 10% FBS for the indicated time period. P493-6 cell line was kindly provided by professor P. Tassone (University “Magna Græcia” of Catanzaro).

pLenti-CNT and pLenti-IBTK-Knockut (KO) HeLa cells were generated through infection with pLenti-CRISPR v2-based lentiviral plasmids. In brief, HEK293T cells were grown in DMEM medium, supplemented with 10% FBS. At a confluence of ~ 50%, cells were transfected using calcium-phosphate transfection method with pLenti-CRISPR v2 (10 μg), pCMV-dR8.1 (10 μg) and pCMV-VSV-G (5 μg) vectors. The day after transfection, the medium was removed and cells replenished with fresh DMEM, containing 3% FBS. At 48 h post transfection, cell medium was collected, filtered through 0.22 μ sterile filter, and used to perform spinoculation in presence of 8 μg/ml polybrene. After 48 h, infected HeLa cells were subjected to selection with puromycin at a final concentration of 5 μg/ml. Transient transfection of siRNA Myc was performed using Lipofectamine 2000 (Life Technologies), according to the manufacturer’s protocol. In brief, cells (3 × 10^6^) were transfected with 100 nmol of the indicated siRNA. RNA interference for *myc* was carried out by using the TranSilent Human *myc* small interfering RNA (siRNA) (Cat#4392420 s9129; Ambion, Thermo Fisher Scientific). Non-targeting scrambled negative control siRNA (Cat#AM4611; Ambion, Thermo Fisher Scientific) was used as a negative control.

### CRISPR/Cas9 vector generation

The gRNA targeting human IBTK was designed using the Optimized CRISPR Design tool (crispr.mit.edu). We scanned the first 10 exons of IBTK gene and we found a guide potentially targeting human *IBTK* for KO, located on chr6: + 82933811, exon 7, with a quality score of 92 and 69 potentially off-target sites (six in genes). Finally, the guide sequence (ACAATCATTGGCGTTGCAGCAGG) was cloned in the vector lentiCRISPR v2 was a gift from Feng Zhang (Addgene plasmid # 52961) following Zhang protocol^25,26^.

### Real-time PCR

Total RNA was extracted from cells using TRIzol reagent (Thermo Fisher Scientific) according to manufacturer’s protocol.

Real-Time PCR was performed with the PowerUP Sybr green master mix (Thermo Fisher Scientific) using a Quant Studio 7 Flex instrument and Fast gene-expression method: 95 °C, 20”; (95 °C, 1”; 60 °C, 20”) × 40 cycles; 95 °C, 15”; 60 °C 1′; 0.05 °C/s up to 95 °C. Real-Time data were analyzed using Quant Studio Real-Time PCR Software (Thermo Fisher Scientific). Reactions were carried out in triplicate, and gene-expression levels were calculated relatively to β-Actin mRNA levels as endogenous control. Real-Time PCR amplification values were reported as 2^−ΔCt^, were ΔCt is Ct_gene_ under investigation−Ct_endogenous control_^27^.

The following primers were used: for murine *Ibtk* gene are 5′-CCTCCTGTTGTGGATCTCAGAACTAT-3′ and 5′- GAGAAAGTTTAACTCCATGAGAAAC-3′ (100 bp products), murine *myc* gene are 5′-ATTTCCTTTGGGCGTTGGA-3′ and 5′- TCCTGTTGGTGAAGTTCACGTT-3′ (69 bp products).

### Immunophenotyping

Grinding and filtering tissues through 0.4 μm cell strainers (BD Biosciences, San Jose, CA, USA) in phosphate-buffered saline (PBS) obtained single-cell suspensions from lymphomas, bone marrow (BM) and spleen of pre-cancerous mice. The suspension was transferred to a fresh tube for centrifugation at 1000 × *g* for 5 min. Pellet was depleted of erythrocytes by lysis with red blood cell lysis buffer (Ammonium chloride–Potassium Lysing Buffer, ACK solution, Lonza, Warkersville, MD, USA), incubated for 1 min at room temperature, suspended in PBS and centrifuged for 1000 × *g* for 5 min. Cell aliquots were first stained with anti-mouse IgM biotin-labeled (dilution 1:100 in PBS) for 15 min at 4 °C in the dark, and centrifuged for 1000 × *g* for 5 min; then, were incubated with anti-mouse CD19 APC-labeled, anti-mouse B220(CD45R)FITC-labeled, anti-mouse IgD PE-labeled and Streptavidin APC/Cy7-labeled (BD Biosciences) (dilution 1:100 in PBS; BD Biosciences, USA) for 15 min at 4 °C in the dark, washed, and suspended in Cytofix-Cytoperm (BD Biosciences) for 15 min at 4 °C in the dark. Consecutively, cell suspension was centrifuged for 1000 × *g* for 5 min and analyzed by flow cytometry. Data were collected by flow cytometer (BriCyteE6, Mindray Bio-Medical Electronics Co. Ltd, Shenzhen, China) and analyzed using FlowJo Version 10.1 software.

### Intracellular flow cytometry

The protocol was modified from Albano, et al.^9^. In brief, single-cell suspensions from BM were incubated with anti-CD19 PE-labeled and anti-B220(CD45R) FITC-labeled antibodies, fixed with 4% Paraformaldehyde and permealized with permeabilization buffer (BD Biosciences). The cells were incubated with anti-IBTK antibody (Novus NBP1-88512) in PBS, 3% fetal bovine serum followed by anti-rabbit-APC antibody (SouthernBiotech 4050- 11 S) for 30 min at room temperature. The mean fluorescence intensity (MFI) was measured by flow cytometry.

### In vitro proliferation assays

Cells were treated with CellTrace CFSE (cell proliferation kit, Thermo Fisher Scientific) at a final concentration of 5 μm for 20 min at 37 °C. Labeling was blocked by adding five volumes of culture medium containing 10% FBS. Cells (1 × 10^6^ cells/ml) were cultured in complete medium. Cell proliferation was calculated by monitoring the decrease in fluorescence label intensity in successive daughter cell generations^28^. Two hours later (Time 0) and after 24 or 48 h, the MFI was measured by flow cytometry. Percentage of fluorescence relative to the starting point was calculated.

### Cell viability and cell death assays

Viability of premalignant B-lymphoid cells from BM and spleen was determined by Trypan Blue Dye exclusion and CellTiter-Glo® Luminescent Cell Viability Assay (Promega, Madison, WI, USA), based on quantitation of ATP, an indicator of metabolically active cells, according to the manufacturer’s instructions. For apoptotic assay, cells were stained with FITC-conjugated Annexin V and propidium iodide (PI) using the Annexin V-FITC kit (Miltenyi Biotech). Data were collected by flow cytometry. Caspase-Glo® 3/7 Assay (Promega) was used to determine the Caspase 3/7 cleavage, according to the manufacturer’s instructions.

### Cell cycle analysis

Cell cycle analysis was performed as previously described^29^. In brief, cells were fixed with 70% (v/v) cold ethanol and stored at –20°C for 1 h. Then, cells were washed with cold PBS, centrifuged and the pellets were resuspended in 200 μL of a non-lysis solution containing 50 μg/mL PI and RNase 250 μg/mL. After incubation at 4° for 30 min, cells were analyzed with flow cytometer (BriCyteE6).

### Western blot analysis

Cells and tissues were lysed in ice-cold modified RIPA buffer (10 mm Tris-HCl, pH 7.5, 150 mm NaCl, 1 mm EDTA, 1% Igepal), as previously described^30^. Protein samples were subjected to electrophoresis on NuPAGE 4–12% polyacrylamide gel (Thermo Fisher Scientific) and then transferred onto a nitrocellulose membrane (BioRad, CA, USA). Equal amounts of protein were Western blotted using the following antibodies: IBtk (#A303-001A; Bethyl Laboratories, Inc., Montgomery, TX, USA), GAPDH (#sc-47724; Santa-Cruz Biotechnology, Dallas, TX, USA), p53 (#sc-393031; Santa-Cruz Biotechnology), Bcl-X_L_ (#2762, Cell Signaling Technology), c-Myc (#5605, Cell Signaling Technology), Bcl-2 (#7382, Santa-Cruz Biotechnology), Bim (#2933, Cell Signaling Technology), Mcl-1 (#D35A5 Cell Signaling Technology), p19ARF (#sc-32748; Santa-Cruz Biotechnology), Vinculin (V9131, Sigma-Aldrich).

### Statistical analysis

Statistical analysis was performed by the two-tailed unpaired Student’s *t* test using GraphPad Prism® software package. Statistical significance was determined by *p* < 0.05. Comparative statistical analysis of Kaplan–Meier survival curves was performed by the Mantel–Cox, Log-rank test.

## Results

### Generation and characterization of Ibtk knockout mice

To address the relevance of *Ibtk* gene we generated an *Ibtk*-deficient mice model by gene-trapping strategy^22^. In mutant XF224 ES cells the gene trap vector inserted *βgeo* reporter gene within the intron 22 of the *Ibtk* gene (Supplementary Fig. 1A). Chimeric mice were generated by microinjection of XF224 ES cells into C57BL/6 J blastocysts, and the derived males were mated with females of C57BL/6 J strain to produce *Ibtk*^*+/−*^ mice, which were inter-crossed to generate *Ibtk*^*−/−*^ littermates. The correct insertion of *βgeo* cassette was verified by PCR of genomic DNA extracted from tails of *Ibtk*^*−/−*^ mice (Supplementary Fig. 1B). The IBtk protein was absent in spleen extracts of *Ibtk*^*−/−*^ mice as shown by Western blotting analysis (Supplementary Fig. 1C). *Ibtk*^*−/−*^ mice were viable and fertile, did not show any gross anatomical defect, and survived as long as *Ibtk*^*+/+*^ mice without developing diseases, including tumors.

Analysis of BM and spleen from mice lacking *Ibtk* revealed no significant defects in B-cell development (Table 1). Peripheral blood cell composition was also unaffected by the absence of *Ibtk* as compared with wild-type control littermates (Table 2).

**Table 1:** Analysis of B-cell subpopulations in *Ibtk*^+/+^ and *Ibtk*^−/−^ mice

	Phenotype	*Ibtk* ^*+/+*^	*Ibtk* ^*−/−*^	*P* value
		Mean(x10^6^) ± SEM	Mean(x10^6^) ± SEM	*Ibtk*^*+/+*^ vs *Ibtk*^*−/−*^
Bone marrow	CD19^+^B220^+^	8.374 ± 1.164	11.32 ± 1.068	0.0921
	CD19^+^B220^low^IgM^-^IgD^-^	5.547 ± 0.9036	8.568 ± 1.182	0.0688
	CD19^+^B220^low^IgM^+^IgD^-^	7.737 ± 1.260	5.838 ± 0.953	0.2892
	CD19^+^B220^hi^IgM^+^IgD^+^	1.210 ± 0.8224	0.8224 ± 0.2798	0.3001
	CD19^+^B220^+^	17.33 ± 2.906	15.60 ± 2.600	0.7000
Spleen	CD19^+^B220^low^IgM^-^IgD^-^	2.040 ± 0.7067	1.628 ± 0.4089	0.6243
	CD19^+^B220^low^IgM^+^IgD^-^	4.546 ± 0.6510	3.190 ± 0.4001	0.1264
	CD19^+^B220^hi^IgM^+^IgD^+^	7.787 ± 1.096	6.440 ± 1.005	0.4064

The absolute number ± SEM of B-lymphoid cells collected from bone marrow and spleen from the indicated genotype (*n* = 6/genotype, age = 3–6 months) is reported

**Table 2:** Peripheral blood cell count in *Ibtk* ^−/−^ and *Ibtk*^+/+^ mice

	*Ibtk* ^*+/+*^	*Ibtk* ^*−/−*^	*P* value
	Mean ± SEM	Mean ± SEM	*Ibtk*^*+/+*^vs *Ibtk*^*−/−*^
WBC (×10^3^ cell/uL)	6.612 ± 0.9303	4.770 ± 0.3226	0.0797
Lymphocytes (×10^3^ cell/uL)	5.855 ± 0.6240	4.714 ± 0.4957	0.1694
RBC (×10^6^ cell/uL)	9.610 ± 0.1507	9.389 ± 0.2789	0.4955
Platelets (×10^3^ cell/uL)	1012 ± 96.66	1205 ± 122.6	0.2510

Number of peripheral blood cells is reported (*n* = 6/genotype, age = 3–6 months)

### Loss of Ibtk delays the onset of pre-B/ B lymphoma in Eμ-myc transgenic mice

Overexpression of Myc is thought to be an initiating event in the development of some B-cell lymphomas. We sought to determine the contribution of *Ibtk* to Myc-induced B-cell lymphomagenesis starting to inter-cross *Ibtk*^−/−^ mice with congenic *Eμ-myc* transgenic mice to generate *Ibtk*^*−/−*^*Eμ-myc* mice. A significant reduction of white blood cells and lymphocytes occurred in peripheral blood of healthy young (4–6 weeks old) pre-cancerous *Ibtk*^−/−^*Eμ-myc* mice compared with *Ibtk*^*+/+*^*Eμ-myc* littermates (Supplementary Fig. 2A, B). No difference was observed in number of platelets and red blood cells (Supplementary Fig. 2C, D).

**Fig. 1:**
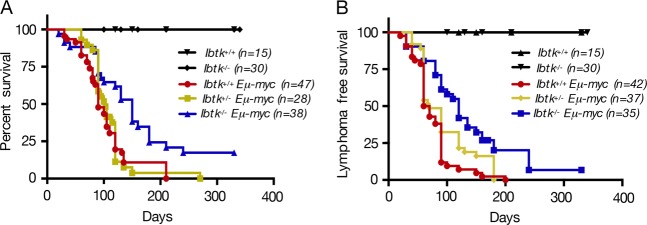
Lack of the *Ibtk* gene increases survival of *Eμ-myc* mice and delays the B-lymphoma onset. **a** Kaplan–Meier survival curves of *Ibtk*^*+/+*^*Eμ-myc, Ibtk*^*+/−*^*Eμ-myc*, and *Ibtk*^*−/−*^*Eμ-myc* mice. Median survival was 90 days for *Ibtk*^*+/+*^*Eμ-myc*, 103 days for *Ibtk*^*+/−*^*Eμ-myc* and 150 days for *Ibtk*^*−/−*^*Eμ-myc* mice. Statistically significance (*p* = 0.0004) was calculated by Mantel–Cox log-rank test. *Ibtk*^+/+^ and *Ibtk*^*−/−*^ mice were included as control. **b** Kaplan–Meier lymphoma-free survival curves of *Ibtk*^*+/+*^*Eμ-myc, Ibtk*^*+/−*^*Eμ-myc* and *Ibtk*^*−/−*^*Eμ-myc* mice. A statistically significant delay of tumor onset occurred in *Ibtk*^*−/−*^*Eμ-myc* mice compared with *Ibtk*^*+/+*^*Eμ-myc* (*p* < 0.0001; Mantel–Cox log-rank test). *Ibtk*^+/+^ and *Ibtk*^*−/−*^ mice were included as control

Littermates were monitored for tumor onset and survival. The lymphoma onset was daily monitored by visual enlargement of one or more peripheral lymph nodes until the time of death. The median age of mortality of *Ibtk*^*+/+*^*Eμ-myc* and *Ibtk*^*+/−*^*Eμ-myc* mice was 90 and 103 days, respectively, with 100% penetrance of lymphoma (Fig. 1a). The lifespan of *Ibtk*^−/−^*Eμ-myc* mice was significantly increased compared with *Ibtk*^*+/+*^*Eμ-myc* (*p* = 0.0004) and with *Ibtk*^*+/−*^*Eμ-myc* (*p* = 0.001) littermates with a median age of mortality of 150 days and 81.6% penetrance of lymphomas (Fig. 1a). The median age of tumor onset was 65 days for *Ibtk*^*+/+*^*Eμ-myc* and 120 days for *Ibtk*^−/−^*Eμ-myc* mice, indicating a statistically significant delay of lymphomagenesis in absence of *Ibtk* (*p* < 0.0001) (Fig. 1b). Loss of a single allele of *Ibtk* did not significantly affect the tumor onset in *Eμ-myc* mice with a median age of 70 days (*p* = 0.06).

Tumor immunophenotyping was performed by flow cytometry using B220, IgM, and IgD as markers of B-cell subpopulations (Supplementary Fig. 3). According to previous reports^10^^,21,31^, 60% pre-B lymphoma (B220^+^IgM^−^IgD^−^), 35% of mature B lymphoma (B220^+^IgM^+^IgD^+^) and 5% pre-B/B lymphomas in *Ibtk*^*+/+*^*Eμ-myc* mice (Table 3). A slight increase of pre-B lymphoma (67%) and pre-B/B lymphoma (11%), with decrease of mature B lymphoma (22%) was observed in *Ibtk*^−/−^*Eμ-myc* mice (Table 3).

**Table 3:** Immunophenotype of lymphomas derived from *Ibtk*^*+/+*^*Eμ-myc* and *Ibtk*^*−/−*^*Eμ-my*c mice

Genotype	Pre-B lymphoma	Pre-B/B lymphoma	B lymphoma
*Ibtk*^*+/+*^ *Em-myc* (*n* = 20)	12 (60%)	1 (5%)	7 (35%)
*Ibtk*^*−/−*^ *Em-myc* (*n* = 18)	12 (66.6%)	2 (11.1%)	4 (22.2%)

Cell suspensions from lymphomas were stained with the antibodies against B220, IgM, and IgD, and analyzed by flow cytometry

These results suggested that the loss of *Ibtk* delayed the onset of Myc-driven lymphoma without significant impact on tumor immunophenotype.

### IBTK expression is regulated by Myc

**Fig. 2:**
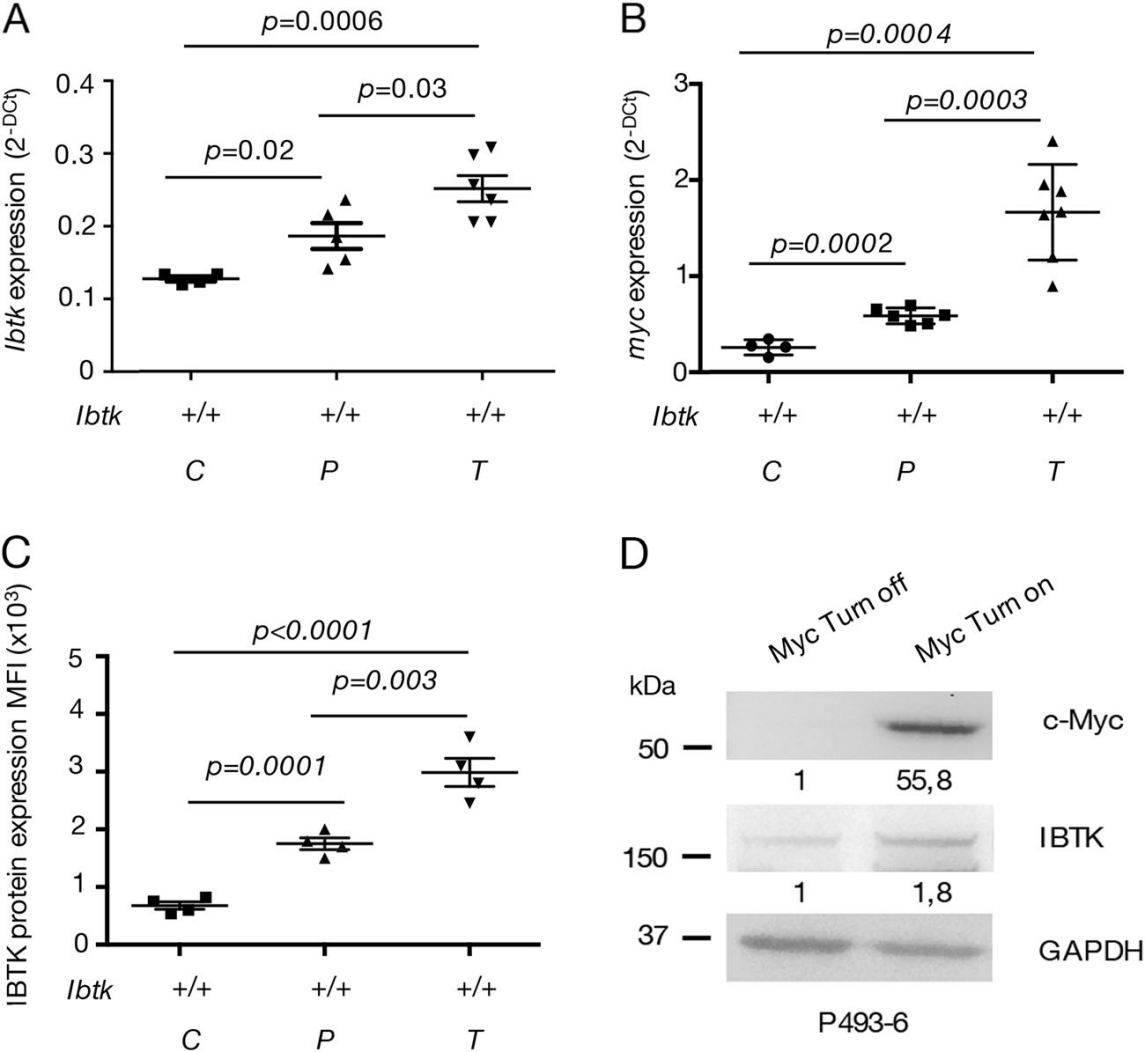
IBTK and Myc expression in B cells. **a** *Ibtk* mRNA levels normalized to the *actin* mRNA in control (C), pre-tumoral (P), and *Eμ-myc* tumors (T) of the reported *Ibtk* genotype, as assessed by RT-qPCR. **b** *myc* mRNA levels normalized to the *actin* mRNA in control (C), pre-tumoral (P), and *Eμ-myc* tumors (T) of the reported *Ibtk* genotype, as assessed by RT-qPCR. **c** The IBTK protein was detected by flow cytometry following the staining of CD19^+^ B220^+^ B cells from BM of control (C), pre-tumoral (P), and *Eμ-myc* tumors (T) with anti-IBtk antibody. The values represent MFI ± SEM (*n* = 4 per genotype). **d** Western blot shows Myc and IBTK protein expression levels in tet-treated (Myc Turn-off) and untreated (Myc Turn-on) P493-6 cells

We proceeded to investigate the expression of IBTK in the *Eµ-myc* mice. Evaluation of *Ibtk* mRNA by quantitative real-time PCR during lymphoma development in murine B cells, showed that *Ibtk* mRNA levels progressively increased (Fig. 2a). Concomitantly, we observed a similar progression of *myc* mRNA levels in the same mice (Fig. 2b), thus proving a direct correlation between the expression levels of the two genes in young non-transgenic, pre-cancerous *Eμ-myc* transgenic littermates and in cancerous Eμ-myc mice. By FACS, we labeling Cd19^+^ B220^+^ cells from BM and observed a significant increase of IBTK protein during lymphomagenesis (Fig. 2c). We took advantage of the P493-6 human B-cell line as a model to examine the impact of Myc on IBTK expression. P493-6 cells bear a tetracycline (tet)-repressible Myc construct such that tet withdrawal results in rapid induction of Myc followed by cell proliferation^32^. Analogous to the results we obtained in pre-cancerous *Eμ-myc* mice, activation of Myc in P493-6 cells increased IBTK levels. In particular, we compared expression levels of Myc and IBTK in tet-treated (Myc turn-off) and untreated (Myc turn-on) cells and observed a direct correlation between IBTK and Myc expression (Fig. 2d). Previous studies suggested that Myc regulated the expression of Ibtk during lymphomagenesis in vivo^33–35^. In particular, Sabò et colleagues^33^ analyzed the genomic distribution of Myc during B-cell lymphomagenesis in the *Eμ-myc* transgenic mouse model. They generated ChIP-sequencing (ChIP-seq) profiles in B cells from young non-transgenic and *Eμ-myc* transgenic littermates and in lymphomas arising in adult *Eμ-myc* animals. Among other genes, they observed that Myc associated with regulatory elements of the *Ibtk* gene. Remarkably, Myc binding intensity of *Ibtk* promoter, increased during lymphoma progression^33^. To validate these data, we confirmed Myc binding on *Ibtk* promoter by evaluation of publically available Cistrome MYC ChIP-seq data^34^ on primary lymphoma B-cell from murine models^32,35,36^ (Supplementary Fig. 4).

In order to identify if *Ibtk* gene is potentially regulated by Myc as a general mechanism, we extended the analysis of Myc binding on *Ibtk* promoter in hematopoietic or nonhematopoietic human cell lines through the evaluation of publically available data set of ChIP-seq experiments^34^. At a genome-wide level, we observed the presence of Myc at the *Ibtk* promoter region in all of which cell lines analyzed (Supplementary Fig. 5). Therefore, we postulated that Myc may be directly modulating *Ibtk* expression.

### Loss of Ibtk decreases the number B-lymphoid cells in pre-cancerous Eμ-myc mice

**Fig. 3:**
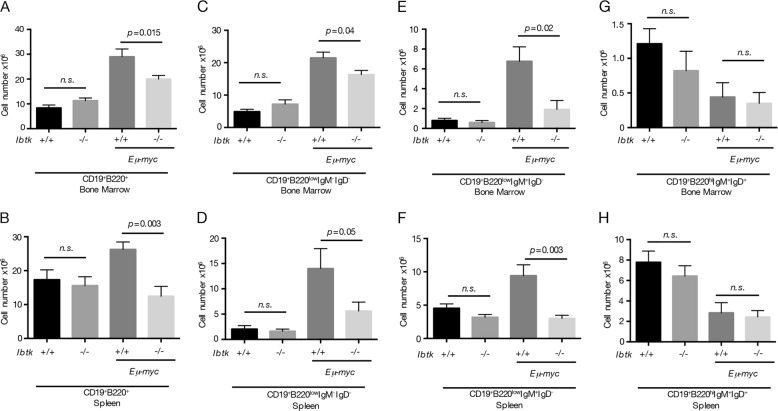
Analysis of B-cell subpopulations in bone marrow and spleen of mice. B cells were magnetically isolated from bone marrow and spleen of healthy (4–6 weeks old) premalignant mice of the indicated genotype, stained with antibodies against CD19 B220, IgM, and IgD, and analyzed by flow cytometry. **a** Absolute numbers of total B cells (CD19^+^B220^+^), **c** pre-B cells (CD19^+^ B220^low^ IgM^−^ IgD^−^, **e** immature B cells (CD19^+^ B220^low^ IgM^+^ IgD^−^), and **g** mature B cells (CD19^+^ B220^hi^ IgM^+^ IgD^+^) of bone marrow are shown (*n* = 6/genotype). **b** Absolute numbers of total B cells (CD19^+^B220^+^), **d** pre-B cells (CD19^+^ B220^low^ IgM^−^ IgD^−^), **f** immature B cells (CD19^+^ B220^low^ IgM^+^ IgD^−^), and **h** mature B cells (CD19^+^ B220^hi^ IgM^+^ IgD^+^) of spleen are shown (*n* = 6/genotype). Values represent the mean ± SEM. Statistically significant difference was evaluated by Student’s *t* test; ns = not statistically significant

A typical signature of Myc-induced B-lymphomagenesis is the aberrant expansion of pre-cancerous immature B-cell population in BM and spleen, accompanied by reduced differentiation to mature B cells^20^^,37^. Thus, it was critical to assess whether the loss of *Ibtk* affected the early stages of B-lymphomagenesis in pre-cancerous mice. Immunophenotype of cell suspensions from BM and spleen showed that the number of total (CD19^+^B220^+^) B-lymphoid cells did not differ in *Ibtk*^+/+^ and *Ibtk*^*−/−*^ mice, whereas it was reduced in *Ibtk*^−/−^*Eμ-myc* compared with *Ibtk*^*+/+*^ *Eμ-myc* transgenic mice (Fig. 3a, b). These results indicate that the delayed onset of B-lymphomagenesis in *Ibtk*^−/−^ *Eμ-myc* mice may be owing to the phenomena that a reduction in *Ibtk* diminishes the Myc-driven expansion of pre-cancerous total B cells. Noteworthy, pre-B (CD19^+^ B220^low^ IgM^−^ IgD^−^) and immature (CD19^+^ B220^low^ IgM^+^ IgD^−^) B cells in BM and spleen were significantly reduced in pre-cancerous *Ibtk*^−/−^ *Eμ-myc* compared with *Ibtk*^*+/+*^ *Eμ-myc* littermates (Fig. 3c–f). Consistent with previous studies^10,38^, the number of mature B cells was reduced in BM and spleen of *Eμ-myc* mice compared with wild-type controls. No statistically significant difference was observed in the number of mature B (CD19^+^ B220^hi^ IgM^+^ IgD^+^) cells in *Ibtk*^−/−^ *Eμ-myc* compared with *Ibtk*^*+/+*^ *Eμ-myc* littermates. (Fig. 3g, h). Altogether these results indicated that *Ibtk* deficiency delayed the onset of Myc-induced lymphoma by reducing the number of pre-B and immature B cells at the pre-cancerous stage.

### Loss of Ibtk impairs the viability of pre-cancerous Eμ-myc B cells by increasing apoptosis

**Fig. 4:**
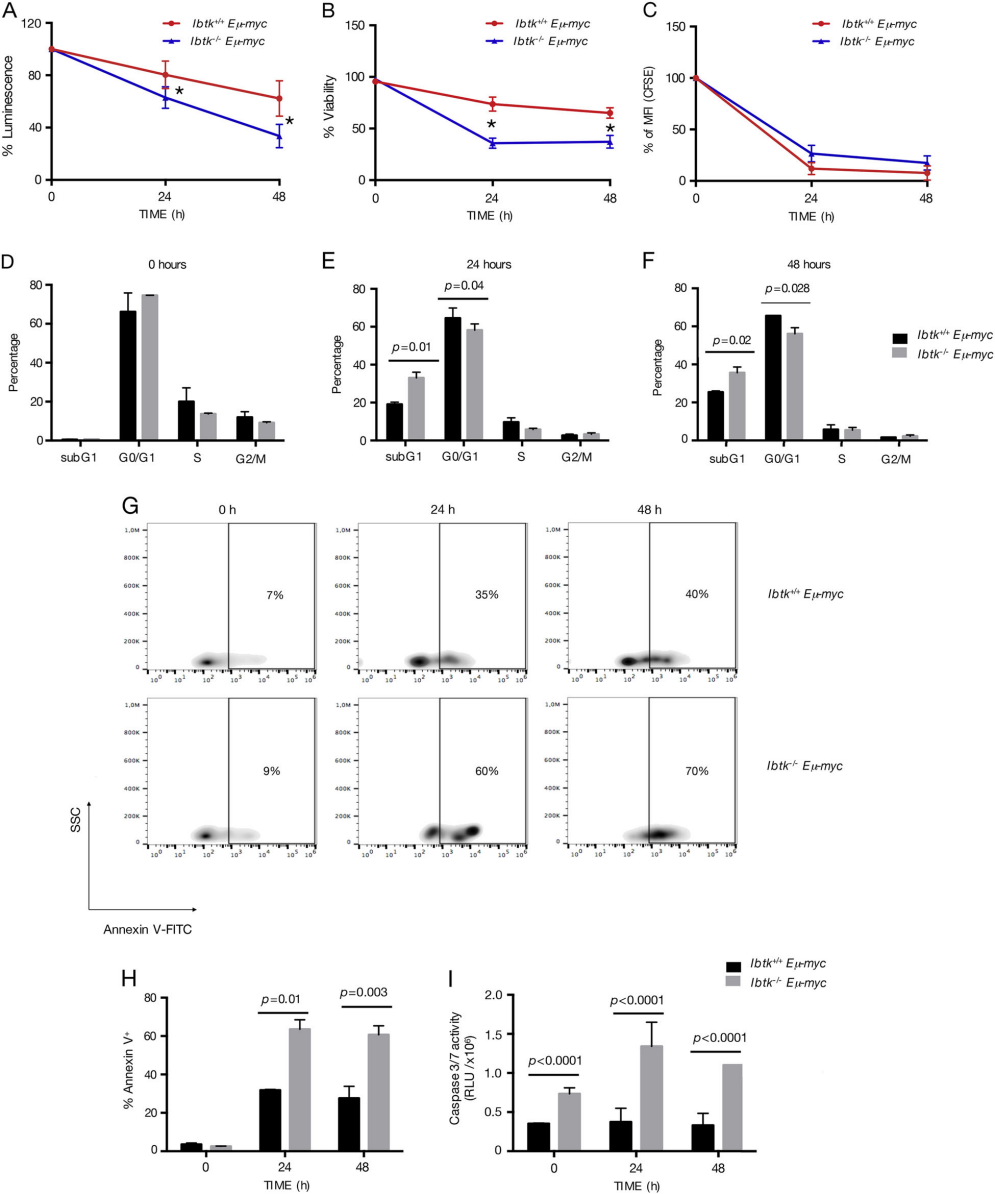
Loss of *Ibtk* reduces viability and increases apoptosis of premalignant pre-B cells. Pre-B cells were isolated from bone marrow of 4–6 weeks old mice of the indicated genotypes using magnetic beads coated with CD19 antibody. Cells were in vitro cultured until 48 h in conventional medium without additional cytokines. Viability was determined at the indicated time points, in technical triplicate by CellTiter-Glo assay **a** and Trypan Blue Dye exclusion assay **b**, as reported in Materials and Methods. Data shown are representative results of independent experiments from cells isolated from four mice/genotype. The values are shown as percentage referred to time 0. Error bars indicate the SEM. *P < 0.006 by Student’s *t* test. **c** CellTrace CFSE-based proliferation assay of pre-B (B220^+^IgM^−^IgD^−^) cells purified using magnetic beads coated with CD19 antibody from the bone marrow of healthy *Ibtk*^*−/−*^*Eμ-myc* and *Ibtk*^*+/+*^*Eμ-myc* mice in vitro cultured for 48 h. Analysis was performed by flow cytometry. The values represent MFI ± SEM as percentage referred to time 0 (*n* = 4 per genotype). **d–f** Cell cycle analysis was performed at the indicated time points. Cells were labeled, fixed then stained with PI/RNase staining solution, and analyzed by flow cytometry. Cell cycle phases were determined using the Watson pragmatic model within the flow cytometry data analysis software FlowJo Version 10.1. Bars represent mean ± SEM; *n* = 4–5 per genotype. **g** Representative density plot of Annexin V binding assay of in vitro cultured pre-B cells of *Ibtk*^*+/+*^*Eμ-myc* and *Ibtk*^*−/−*^*Eμ-myc* mice. Cells were labeled then gated for pre-B cells (B220^+^IgM^−^) and Annexin V binding was analyzed. **h** Bar diagram showing the quantification of apoptotic cells as mean of three independent experiments ± SEM. **i** Measure of Caspase 3/7 cleavage (in triplicate) was performed. Values are the mean of at least three independent experiments ± SEM

Next, we measured the viability and growth rate of pre-B cells derived from BM. To this end, cells were cultured in the absence of exogenous cytokines for 48 h. The loss of *Ibtk* led to a time-dependent viability of *Eμ-myc* pre-B cells (Fig. 4a, b).

Flow cytometric analysis showed no difference in proliferation rate of *Ibtk*^−/−^*Eμ-myc* mice compared with *Ibtk*^*+/+*^*Eμ-myc*, as measured by CellTrace CFSE staining (Fig. 4c). Further, we analyzed cell cycle using PI staining. There was no difference between *Ibtk*^*+/+*^*Eμ-myc* and *Ibtk*^−/−^*Eμ-myc* pre-cancerous pre-B-cell number in S-phase either before culture (Fig. 4d) or at any of the time points analyzed (Fig. 4e, f). As compared with *Ibtk*^*+/+*^*Eμ-myc*, the number of apoptotic subG1 cells was significantly increased in *Ibtk*^−/−^*Eμ-myc* mice (from 20.2 to 33.07%, *p* value = 0.01; from 25.45% to 35.63%, *p* value = 0.02; time 24 h and 48 h, respectively), along with a decrease of G0/G1 phase at 48 h (from 65.55% to 56.1%, *p* value = 0.028).

Then, we proceeded to confirm that the loss of *Ibtk* influences apoptosis of Myc-overexpressing B cells. Of note, increased apoptosis spontaneously occurred in pre-cancerous pre-B cells isolated from BM of *Ibtk*^−/−^*Eμ-myc* mice compared with *Ibtk*^*+/+*^*Eμ-myc* mice, when cultured in simple medium as measured by Annexin V-binding assay (Fig. 4g, h). Increased apoptosis in *Ibtk*^−/−^*Eμ-myc* mice was also confirmed by Caspase 3/7 cleavage assay (Fig. 4i).

**Fig. 5:**
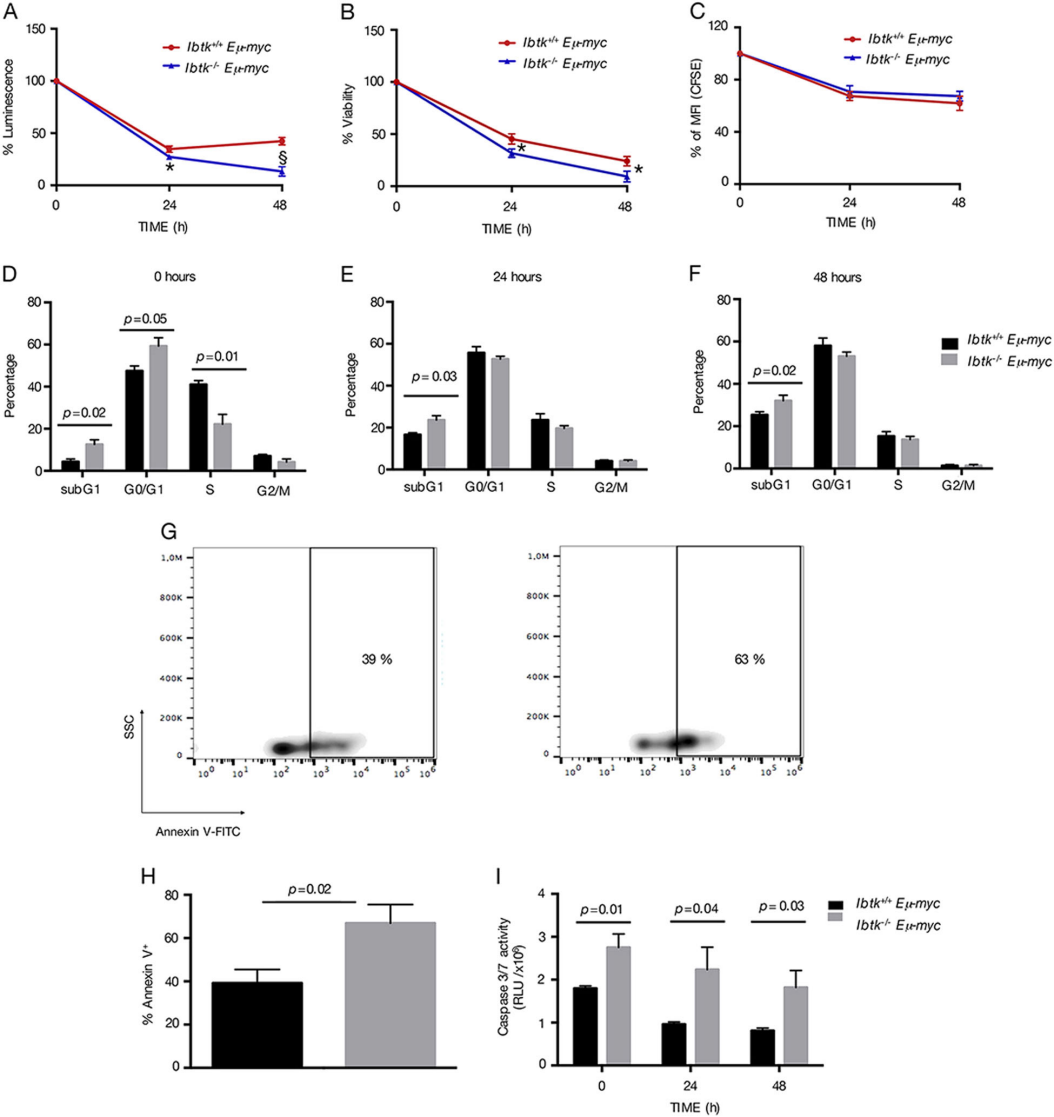
Loss of *Ibtk* reduces viability and increases apoptosis of pre-cancerous splenic B cells. Splenic B-lymphoid cells were in vitro cultured and viability was assessed. Cell viability was measured at 0, 24, and 48 h in technical triplicate by CellTiter-Glo assay **a** and Trypan Blue Dye exclusion **b**., as reported in Materials and Methods. Data shown are representative results of independent experiments from cells isolated from four mice/genotype. The values are shown as percentage referred to time 0. Error bars indicate the SEM. *P < 0.006, and ^§^*P* < 0.0009, by Student’s *t* test. **c** CellTrace CFSE-based proliferation assay of B (B220^+^) cells purified using magnetic beads coated with CD19 antibody from the spleen of healthy *Ibtk*^*−/−*^*Eμ-myc* and *Ibtk*^*+/+*^*Eμ-myc* mice in vitro cultured for 48 h. Analysis was performed by flow cytometry. The values represent MFI ± SEM as percentage referred to time 0 (*n* = 4 per genotype). **d**–**f** Cell cycle at the indicated time points was reported. Cells were fixed then stained with PI/RNase staining solution. Cell cycle phases were determined using the Watson pragmatic model within the flow cytometry data analysis software FlowJo Version 10.1. Bars represent mean ± SEM; *n* = 4–5 per genotype. **g** Representative density plot of Annexin V-binding assay. Apoptotic splenic cells selected at the gate for B220^+^IgM^−^ were stained with V-FITC/PI and analyzed by flow cytometry. **h** Percentage of apoptotic cells is also shown in bar diagram as mean of three independent experiments ± SEM. **i** Measure (in triplicate) of Caspase 3/7 cleavage was performed. Values are the mean of at least three independent experiments ± SEM

Next, we also measured the viability and growth rate of splenic B cells. To this end, we cultured cells for 48 h. The loss of *Ibtk* led to a decrease of viability of *Eμ-myc* B cells (Fig. 5a, b). We also performed proliferation, cell cycle and apoptosis analysis. Flow cytometric analysis showed no difference in proliferation rate of *Ibtk*^−/−^*Eμ-myc* mice compared with *Ibtk*^*+/+*^*Eμ-myc*, as measured by CellTrace CFSE staining (Fig. 5c). In absence of *Ibtk*, cell cycle analysis of splenic B cells showed a persistent increase of apoptotic subG1 population from time 0 up 48 h, with a transient increase of G0/G1 cells and decrease of S cells only at time 0 (Fig. 5d–f).

The higher spontaneous apoptotic rate of splenic B cells from *Ibtk*^−/−^*Eμ-myc* was ex vivo confirmed by Annexin V binding assay (Fig. 5g, h) and by Caspase 3/7 cleavage of in vitro cultured cells (Fig. 5i). These data suggest that the loss of *Ibtk* increased the sensitivity of *Eμ-myc* splenic B cells to apoptosis without affecting their proliferation rate. Altogether, these results indicated that *Ibtk* conferred resistance to apoptosis of pre-cancerous B cells in Myc-driven B lymphoma.

### Loss of Ibtk affects the main Myc-driven apoptosis pathways in pre-cancerous Eμ-myc mice

Myc induces apoptosis by activating different pathways. For instance, by impairing p53 /p19ARF expression and/or by suppressing the expression of anti-apoptotic BCL-2 family members^39–41^.

Deregulated Myc expression can inactivate the p53 tumor-suppressor pathway during lymphomagenesis. About 30% of *Eμ-myc* tumors carry mutations in the p53 pathway^42^ As mutations in p53 typically result in protein overexpression^43^. Inactivation of the p53 pathway can also arise with ARF loss, but it occurs only rarely in *Eμ-myc* mice^42,44^.

**Fig. 6:**
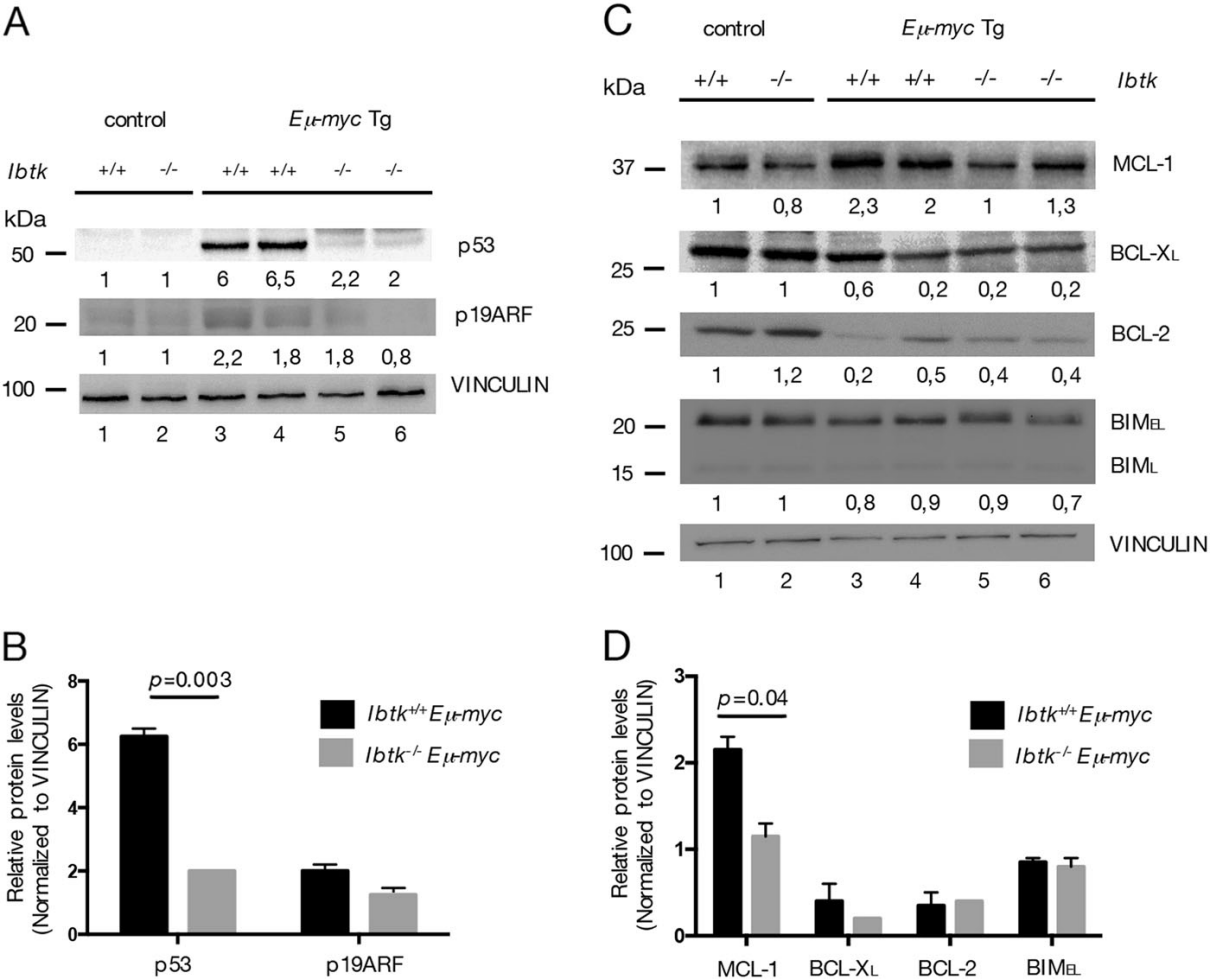
Loss of *Ibtk* affects Bcl-2 and p53/ARF pathways. **a** Immunoblot analysis of p19ARF and p53 in splenic B cells from non-Tg (lanes 1, 2) and premalignant (lanes 3–6) *Ibtk*^*+/+*^*Eμ-myc* and *Ibtk*^*−/−*^*Eμ-myc* mice (total samples analyzed = 32; *n* = from 6 to 10 per genotype). Vinculin is used as loading control. **b** Quantification of p53 and p19ARF levels of experiment described in **a**. Protein bands were normalized to the corresponding Vinculin intensity. The mean densitometric values ± SEM of two independent loaded sample are shown. **c** Immunoblot analysis of Bcl-2 family proteins in splenic B cells from non-Tg (lanes 1, 2) and premalignant (lanes 3–6) *Ibtk*^*+/+*^*Eμ-myc* and *Ibtk*^*−/−*^*Eμ-myc* mice (total samples analyzed = 32; *n* = from 6 to 10 per genotype). Vinculin is used as loading control. **d** Quantification of MCL-1, BCL-2, BCL-X_L_, and BIM levels of experiment described in **b**. Protein bands were normalized to the corresponding Vinculin intensity. The mean densitometric values ± SEM of two independent loaded sample are shown

Consistently with this notion, we performed Immunoblot analysis of splenic B cells from pre-cancerous *Ibtk*^*+/+*^ *Eμ-myc* and littermate *Ibtk*^*−/−*^ *Eμ-myc* mice and littermate-matched non-Tg mice (Fig. 6a). p53 is clearly accumulated in prelymphomatous *Ibtk*^*+/+*^ *Eμ-myc* (4 of 10 samples, 40%) compared with *Ibtk*^*−/−*^ *Eμ-myc* splenic B cells (1 of 10 samples, 10%; Fig. 6a, b). p19ARF protein is detected in prelymphomatous *Ibtk*^*+/+*^ *Eμ-myc* (5 of 10 samples, 50%; Fig. 6a) compared *Ibtk*^*−/−*^ *Eμ-myc* splenic B cells (4 of 10 samples, 40%), without any statistical difference in the expression of p19ARF protein between prelymphomatous *Ibtk*^*+/+*^ *Eμ-myc* and *Ibtk*^*−/−*^ *Eμ-myc* splenic B cells (Fig. 6b). Western blot analysis for p53 and p19ARF proteins revealed that there was a significant difference in the p53/p19ARF pathway between *Ibtk*^*+/+*^ *Eμ-myc* versus *Ibtk*^*−/−*^ *Eμ-myc* mice with an increase of p53 protein in *Ibtk*^*+/+*^ *Eμ-myc* rather to *Ibtk*^*−/−*^ *Eμ-myc* splenic B cells. These results suggest that the elevated p53 levels are owing to Myc-dependent activation of p53 in *Ibtk*^*+/+*^ *Eμ-myc* mice. In *Ibtk*^*−/−*^ *Eμ-myc* B cells, likely, p53 protein levels could be eliminated before reaching high levels of Myc-dependent accumulation (since the loss of *Ibtk* sensitizes these cells to Myc-induced apoptosis).

We also addressed the question whether *Ibtk* affected the levels of BCL-2 family proteins in splenic B cells of non-Tg mice and pre-cancerous *Eμ-myc* mice. In non-Tg mice, the expression level of MCL-1, BCL-X_L_, BCL-2, BIM proteins did not differ in presence or absence of *Ibtk* (Fig. 6c, lanes 1, 2). BCL-2, BCL-X_L_, BIM expression levels resulted unchanged by comparing splenic B cells from *Ibtk*^*+/+*^ *Eμ-myc* with *Ibtk*^*−/−*^ *Eμ-myc* mice (Fig. 6c, d). Interestingly, loss of *Ibtk* significantly decreased the expression of MCL-1 (Fig. 6c, d). Recent evidence highlights a critical role for the BCL-2 family member MCL-1 in several lymphoma subtypes^45^. As previously reported^46,47^, MCL-1 expression was elevated in pre-cancerous *Eμ-myc* cells, particularly in pro/pre-B. Previous genetic studies have shown that the development of B-lymphoid tumors in *Eμ-Myc* mice is critically dependent on expression of pro-survival MCL-1 and it is dispensable for sustained growth of fully malignant lymphoma cells in transplant recipients^47,48^.

Taken together, these observations indicate that IBTK probably impacts on the apoptotic pathway driven to Myc overexpression in pre-cancerous *Eμ-myc* mice, acting both on p53-dependent and p53-independent apoptotic program.

### Loss of Ibtk increases apoptosis and cell cycle arrest in nonhematopoietic human cells independently of Myc

**Fig. 7:**
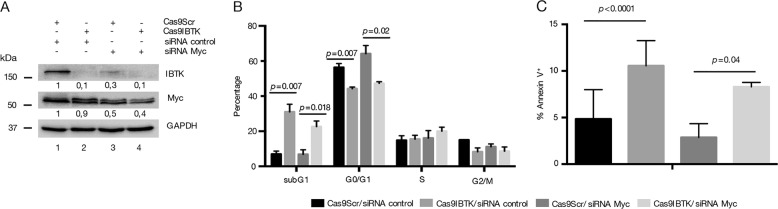
Loss of *Ibtk* impairs cell cycle and increases apoptosis in HeLa cell line independently of Myc. **a** Representative western blot of IBTK and Myc protein expression in HeLa cells knocked down by CRISPR-Cas9 method and silenced by siRNA Myc. **b** Cell cycle phases were determined using the Watson pragmatic model within the flow cytometry data analysis software FlowJo Version 10.1. Bars represent mean ± SEM; Values are the mean of at least three independent experiments ± SEM. **c** Percentage of apoptotic cells is shown in bar diagram as mean of three independent experiments ± SEM

Abnormal activation of Myc is known to contribute to cervix carcinogenesis^49^. Consistent with this notion, we proceeded to analyze whether IBTK could regulate apoptosis in HeLa cell line (cervix cancer cells). We knocked out *IBTK* using CRISPR/Cas9 system. Specifically, we transduced cancer cells with lentiviruses carrying both Cas9 and sgRNA components. We observed a significant depletion of IBTK protein in HeLa^Cas9IBTK^ cells compared with scrambled HeLa^Cas9Scr^ (Fig. 7a). Flow cytometric analysis showed a cell cycle arrest with increased apoptosis in *IBTK-*silenced cells, as showed by cell cycle profiles (Fig. 7b). As compared with HeLa^Cas9Scr^, the number of apoptotic subG1 cells was increased in HeLa^Cas9IBTK^ (from 5.45 to 31%, *p* value = 0.02) along with a decrease of G0/G1 phase (from 54.25% to 44.23%, *p* value = 0.004). Further, increased apoptosis in HeLa^Cas9IBTK^ cells was also confirmed by Annexin V-binding assay (Fig. 7c). Then, we transfected wild-type or IBTK knockdown HeLa cells with siRNA scrambled or siRNA Myc. The observed consequence was a reduction of Myc protein content in HeLa^Cas9Scr^ and HeLa^Cas9IBTK^ silenced with siRNA Myc compared with HeLa^Cas9Scr^ and HeLa^Cas9IBTK^ transfected with siRNA control (Fig. 7a, b).

In agreement with the increased expression of IBtk in P493-6 cells upon the induction of Myc (Fig. 2d), we observed that Myc silencing decreased IBtk expression (Fig. 7a), supporting the hypothesis of Myc-dependent *IBTK* expression.

Consistent with previous studies demonstrating that Myc RNA interference did not impair cell cycle and apoptosis in HeLa cells^49^, we found that Myc depletion did not affect cell cycle distribution or apoptosis cell death (Fig. 7c, d).

Based on our data, in B-cell context IBTK acts as a negative regulator of Myc-dependent apoptosis, whereas in non-B cells, IBTK could affect the apoptosis independently of Myc.

## Discussion

Apoptosis counteracts cell proliferation driven by oncogenes, thus limiting cancer development^50^. In *Eμ-myc* transgenic mouse model of B-lymphomagenesis the pre-cancerous state is characterized by aberrant proliferation of B-lymphoid cells, which is initially offset by pro-apoptotic action of c-Myc^51^. Resistance of pre-cancerous B cells to Myc-induced apoptosis must occur for proceeding toward malignancy. Several mechanisms have been identified to overcome apoptosis of pre-cancerous B cells, including inactivating mutations of ARF and/or p53^42,52^, overexpression of anti-apoptotic Bcl-2 proteins (including BCL-2, BCL-X_L_, Mcl-1, BCL-w)^48,53–55^, and loss of the pro-apoptotic BH3-only proteins Bim^56^, Bmf^57^, or Puma^21^.

In this study, we have shown that the *Ibtk* gene is required for counteracting apoptosis of Myc-driven B cells in *Eμ-myc* mice. Loss of *Ibtk* increased the median age of animal survival and significantly delayed the onset of B-cell lymphoma. *Ibtk*^*−/−*^*Eμ-myc* mice mostly developed pre-B lymphoma and to a lesser extent mature B lymphoma, which was consistent with the tumor phenotype of *Eμ-myc* transgenic mice^10,20^. Loss of *Ibtk* substantially reduced the number of premalignant B-lymphoid cells without affecting their proliferation rate. In particular, pre-cancerous immature B cells (B220^low^) were reduced in BM and spleen of *Ibtk*^−/−^*Eμ-myc* compared with *Ibtk*^*+/+*^*Eμ-myc* mice. Furthermore, our data and others^33,35,36^ have shown that Myc was directly regulating the expression of *Ibtk* in murine B-cell lymphoma and in other cell types based on ChIP-seq data analysis.

*Eμ-myc* lymphomas generally derive from the immature B cells (subset), thus the reduced number of pre-cancerous *Ibtk*^*−/−*^*Eμ-myc* pre-B and immature B cells could explain the enhanced survival and delayed tumor onset of *Ibtk*^−/−^*Eμ-myc*^10,20^.

Apoptosis of pre-cancerous B cells was associated with increased Caspase 3/7 cleavage, which was consistent with previous observations in mouse embryonic fibroblasts, where *IBTK* RNA interference reduced the cell survival with increased activation of Caspase 3/7^5^. Our findings are also consistent with pro-survival activity of the human *IBTK* gene in colorectal cancer cells Ras-dependent signaling^7^. Recently, we have observed that *IBTKα* is overexpressed during CLL progression. Further, IBTKα RNA interference in DeFew and MEC-1 cell lines caused spontaneous apoptosis and up-regulation of anti-apoptotic genes^9^.

Noteworthy, loss of *Ibtk* impairs the main anti/pro-apoptotic proteins dependent on Myc overexpression in pre-cancerous *Eμ-myc* mice, such as MCL-1 protein and p53. In this scenario, we can assert that loss of *Ibtk* is required for Myc-driven apoptosis in B-cell context. Could the loss of *Ibtk* be required for Myc-driven apoptosis in different cell types? Based on this question, we analyzed cell viability in presence or absence of IBTK and Myc using CRISPR/Cas9 and siRNA method in HeLa cell line. IBTK silencing arrested the cells in the G0/G1 phase of cell cycle and increased the number of apoptotic cells independently of Myc presence.

These data offer mechanistic evidence of a link between Myc and IBTK in B-cell context, and, we believe, significantly expand our understanding of Myc-mediated apoptosis.

As Myc is abnormally expressed in a great majority of human cancers^17^, the evidence that *Ibtk* promotes the survival of Myc-driven premalignant B cells, could have general implications for oncogenesis.

The mouse Ibtk protein is highly homolog to human IBtkα and it has been demonstrated to function as a substrate receptor of Cul3-dependent ubiquitin ligase (CRL3^IBTK^)^2^. An effort aimed at the identification of novel CRL3^IBTK^ substrates in cancer B cells would be helpful to clarify the mechanism of action in cancer B cells. It is reasonable to hypothesize that IBtkα could affect both transcriptome and proteome at least by affecting the stability of transcriptional and translational activators and repressors.

In summary, our findings provide the first evidence on a synergistic role of IBTK in Myc-driven B-lymphomagenesis mainly through counteraction of B cells apoptosis.

## Supplementary information

supplemental material
