# Supplementary material for: IBTK contributes to B-cell lymphomagenesis in Eμ-myc transgenic mice conferring resistance to apoptosis
Source: Cell Death Dis. 2019 Apr 11;10(4):320. doi: 10.1038/s41419-019-1557-6 (PMC6459904; doi:10.1038/s41419-019-1557-6)
Supplement: Supplementary file 1 — supplemental material [file 41419_2019_1557_MOESM1_ESM.doc]

**Supplementary Materials**

**Supplemental Figure 1**

**
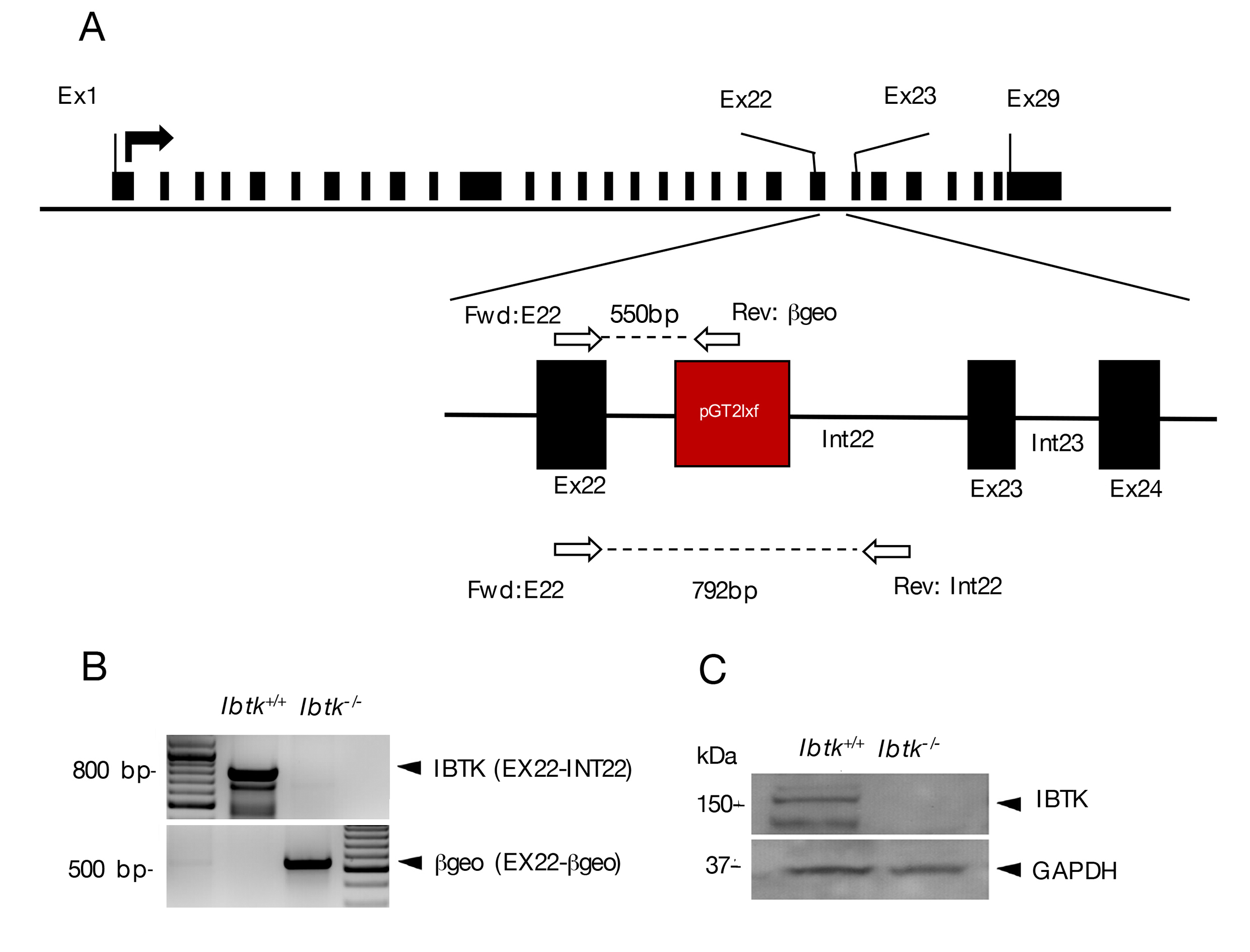
**

**Supplemental Figure 1. Generation of *Ibtk* knock out mice.** (A) Structure of the murine *Ibtk* gene (NC_000075) containing 29 exons. The arrows indicate the annealing site of primers for testing the insert construction. (B) Analysis of genomic DNA of *Ibtk* and *βgeo* genes by PCR. Genomic DNA was extracted from the tail of *Ibtk^+/+^ and* *Ibtk^+/-^* mice. (C) Protein extracts from spleen of *Ibtk^+/+^* and *Ibtk^-/-^* mice were analysed by Western blot using the anti-IBtk Ab (Bethyl Lab).

**Supplemental Figure 2**


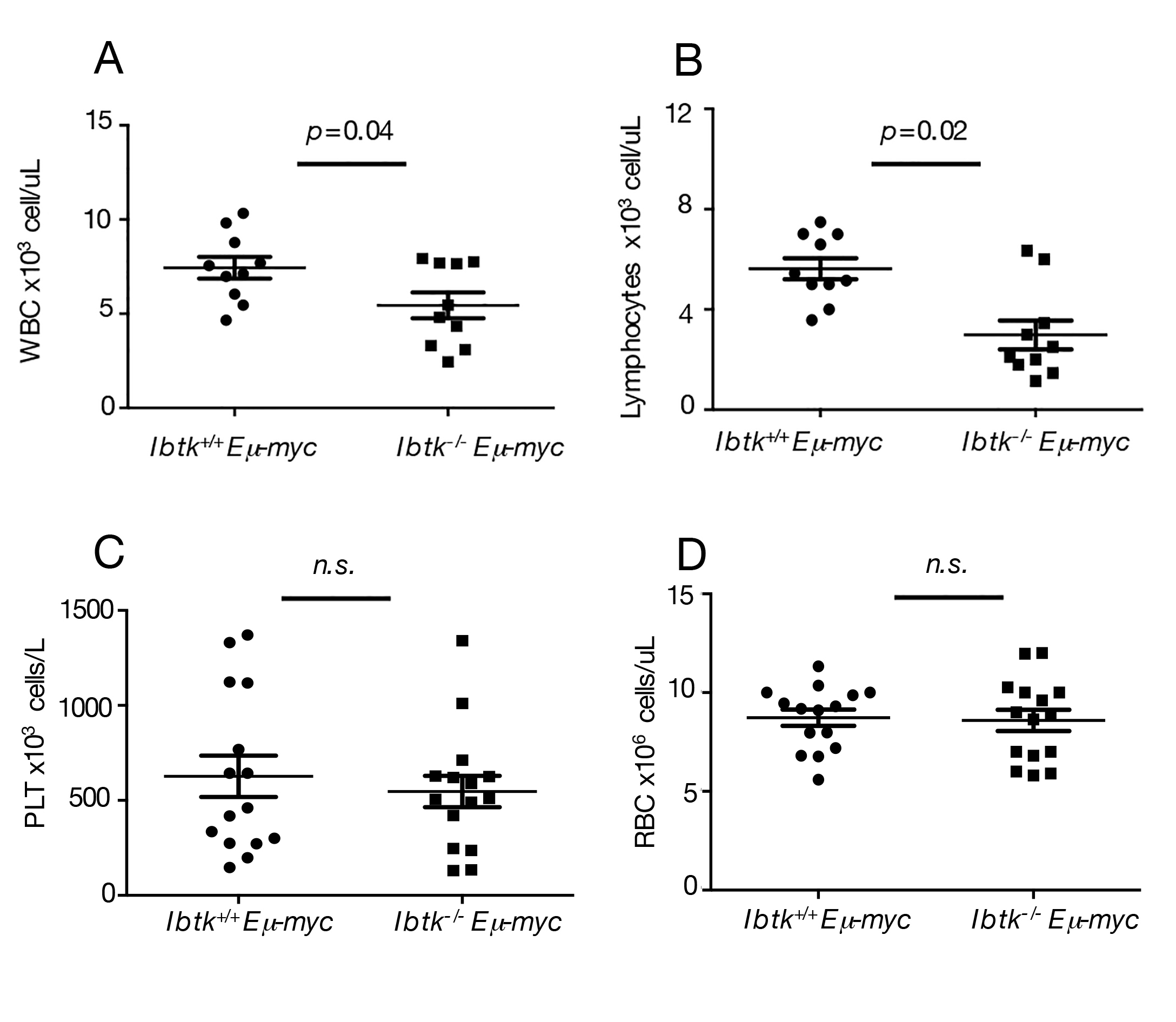


**Supplemental Figure 2. Analysis of peripheral blood of precancerous *Ibtk^+/+^Eμ-myc* and *Ibtk^-/-^Eμ-myc* mice.** (A, B) White blood cells (WBC) (n=10/genotype) and lymphocytes (n=10/genotype) count in peripheral blood of pre-cancerous young and healthy (4-6 weeks old) *Ibtk^+/+^Eμ-myc* and *Ibtk^-/-^Eμ-myc* mice. (C) Number of platelets and (D) red blood cells in peripheral blood is reported (n=15/genotype, 4-6 weeks old). Mean value
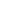
 SEM is indicated. Statistically significant difference was evaluated by Student’s *t* test; ns= not statistically significant.

**Supplemental Figure 3**

**
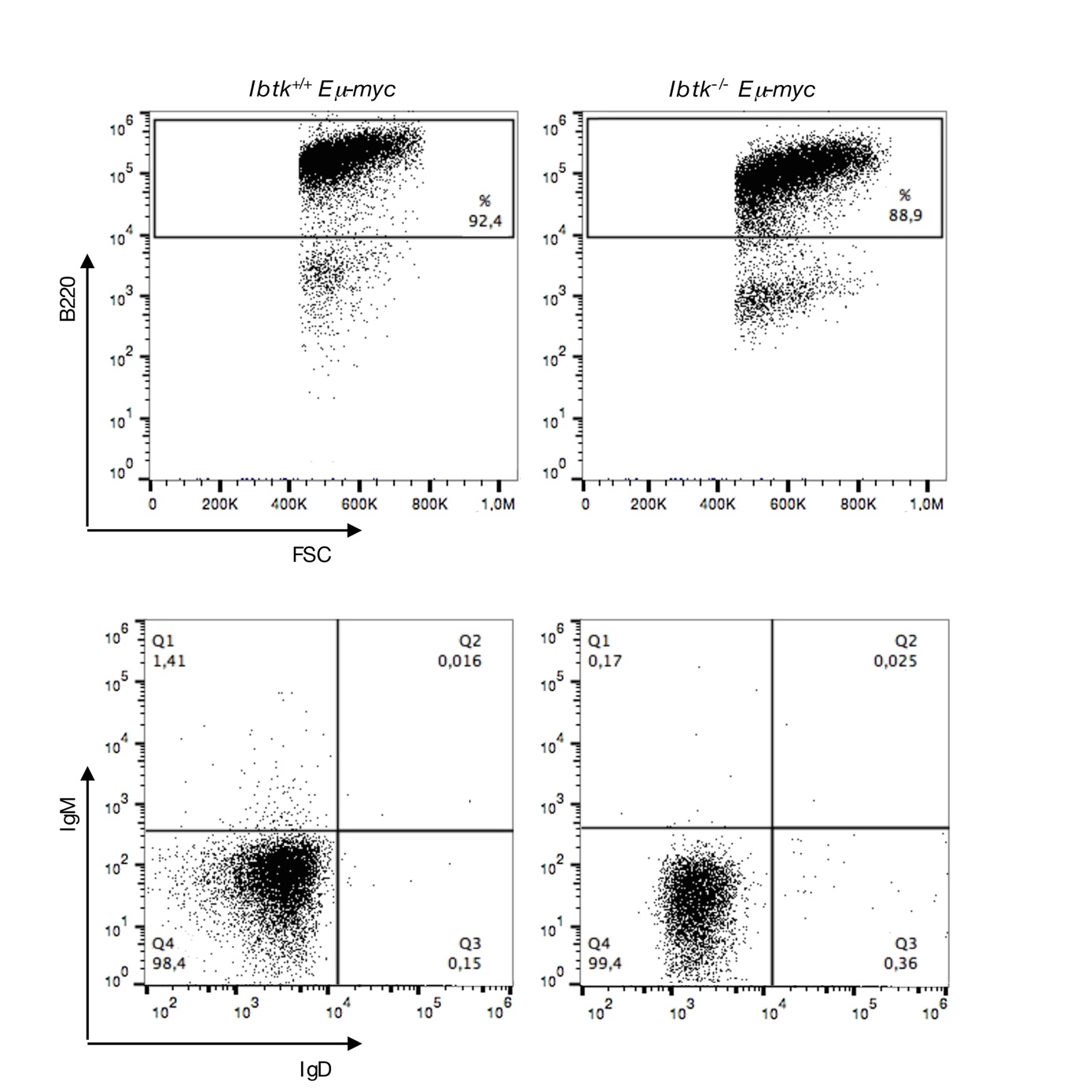
**

**Supplemental Figure 3. The majority of lymphomas derived from *Ibtk^-/-^Em-myc* mice display a sIg^-^ B cell immunophenotype.** Representative immunophenotypic profiles of pre-B lymphoma derived from *Ibtk^-/-^Eμ-my*c and *Ibtk^+/+^Eμ-myc* by flow cytometry using the antibodies against B220, IgM and IgD.

**Supplemental Figure 4**


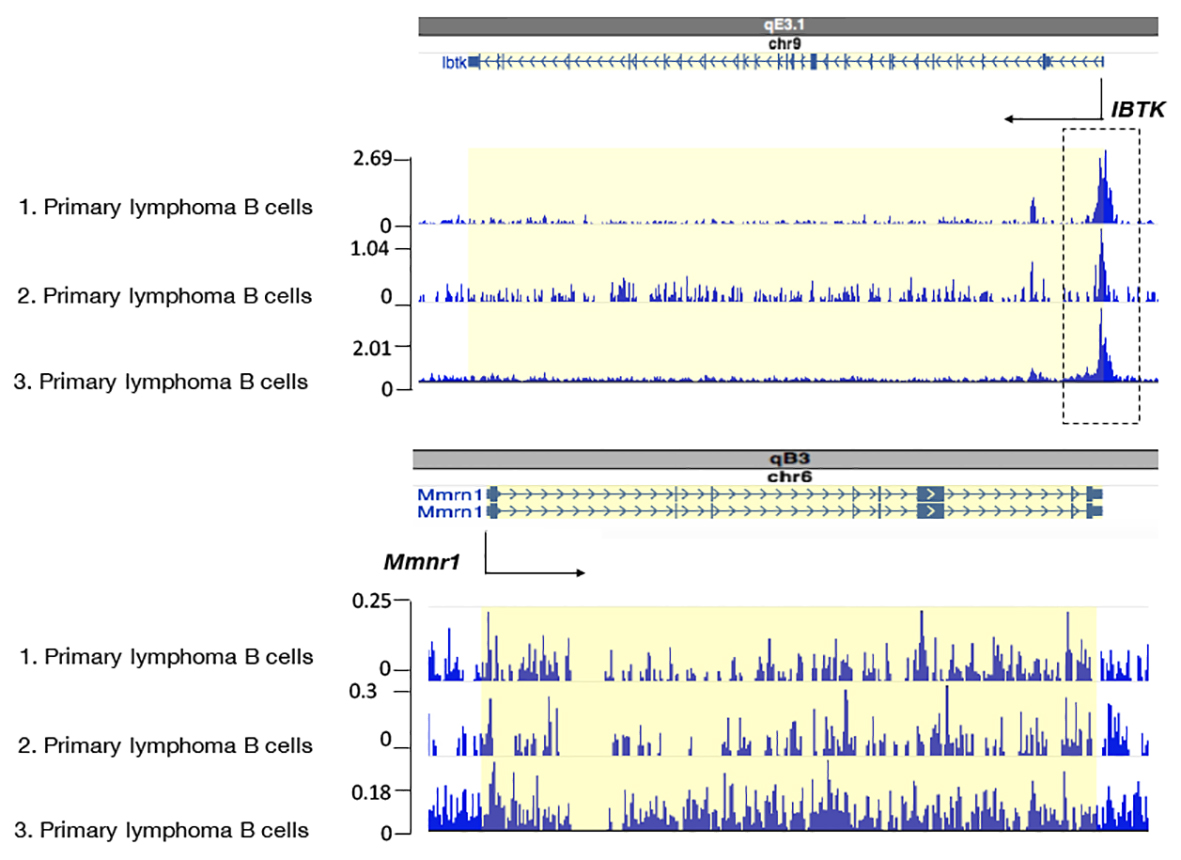


**Supplemental Figure 4**. **MYC is present at the *IBTK* promoter in murine lymphoma B cells.** Evaluation of CISTROME MYC ChIP-seq data demonstrates that MYC is enriched at the *IBTK* promoter region (dashed box) in murine primary Lymphoma B cells. As a negative control, the same data were evaluated for MYC enrichment at the promoter of multimerin-1 precursor (*Mmnr1*), a well-known MYC negative control gene. The peaks represent the fold change over that of the IgG control.

**Supplemental Figure 5**


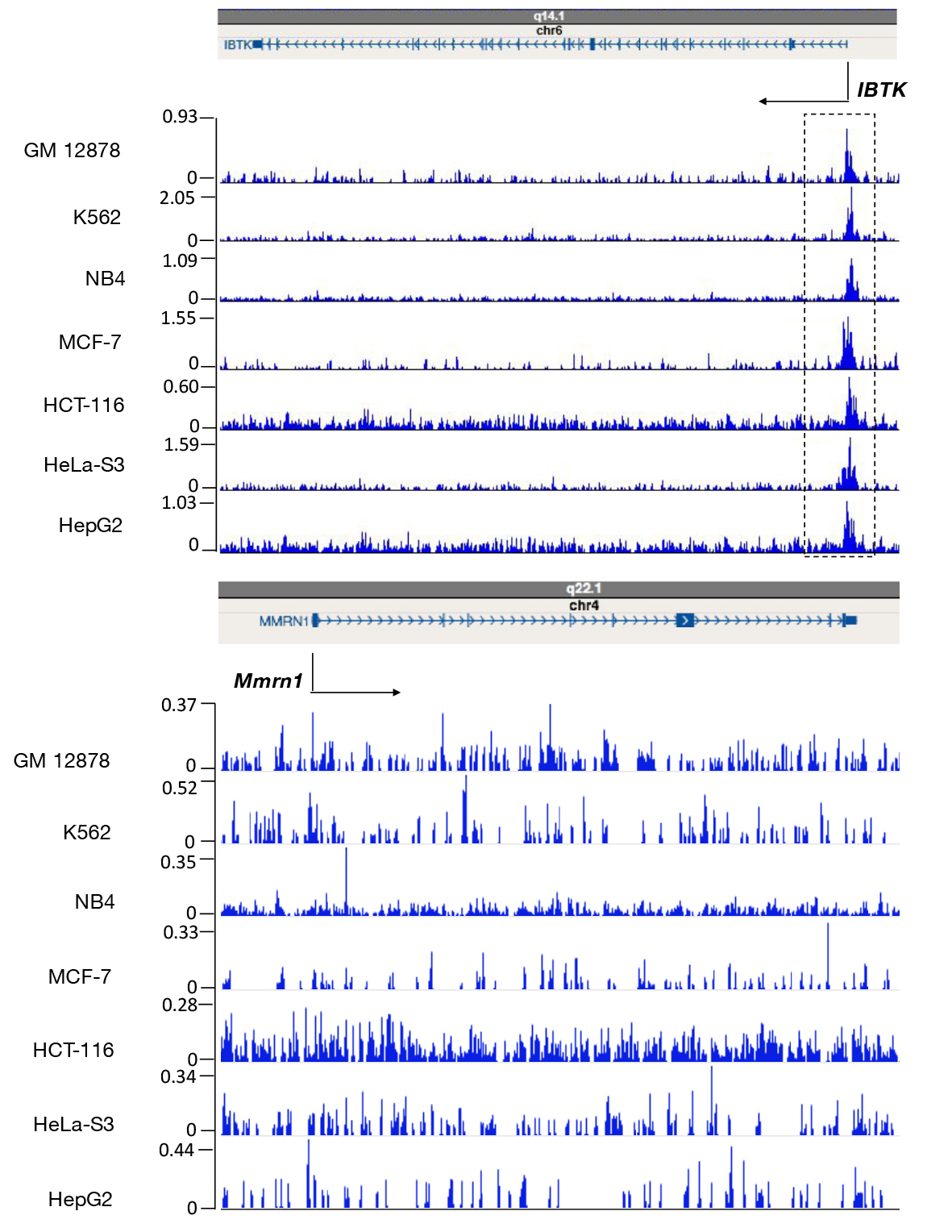


**Supplemental Figure 5**. **MYC is present at the *IBTK* promoter.** Evaluation of CISTROME MYC ChIP-seq data demonstrates that MYC is enriched at the *IBTK* promoter region (dashed box) in multiple human cell lines [chronic myelogenous leukemia (K562), acute promyelocytic leukemia (NB4), lymphoblastoid (GM12878), breast cancer (MCF-7), colorectal carcinoma (HCT-116), cervical carcinoma (HeLa-S3), and hepatocellular carcinoma (HepG2). As a negative control, the same data were evaluated for MYC enrichment at the promoter of multimerin-1 precursor (*Mmnr1*), a well-known MYC negative control gene. The peaks represent the fold change over that of the IgG control.
